# Large field-of-view nanometer-sectioning microscopy by using metal-induced energy transfer and biexponential lifetime analysis

**DOI:** 10.1038/s42003-020-01628-3

**Published:** 2021-01-19

**Authors:** Wonsang Hwang, Jinwon Seo, DongEun Kim, Chang Jun Lee, In-Hong Choi, Kyung-Hwa Yoo, Dug Young Kim

**Affiliations:** 1 Department of Physics, Yonsei University, Seoul, Republic of Korea; 2 Department of Microbiology, Institute for Immunology and Immunological Diseases, College of Medicine, Yonsei University, Seoul, Republic of Korea

**Keywords:** Cellular imaging, Cell adhesion, Nanoscale biophysics, Membrane biophysics

## Abstract

Total internal reflection fluorescence (TIRF) microscopy, which has about 100-nm axial excitation depth, is the method of choice for nanometer-sectioning imaging for decades. Lately, several new imaging techniques, such as variable angle TIRF microscopy, supercritical-angle fluorescence microscopy, and metal-induced energy transfer imaging, have been proposed to enhance the axial resolution of TIRF. However, all of these methods use high numerical aperture (NA) objectives, and measured images inevitably have small field-of-views (FOVs). Small-FOV can be a serious limitation when multiple cells need to be observed. We propose large-FOV nanometer-sectioning microscopy, which breaks the complementary relations between the depth of focus and axial sectioning by using MIET. Large-FOV imaging is achieved with a low-magnification objective, while nanometer-sectioning is realized utilizing metal-induced energy transfer and biexponential fluorescence lifetime analysis. The feasibility of our proposed method was demonstrated by imaging nanometer-scale distances between the basal membrane of human aortic endothelial cells and a substrate.

## Abstract (web summary)

Hwang et al. demonstrate that a high axial resolution can be achieved even with low numerical aperture (NA) objectives. They show the nano-profile of a basal cell membrane using metal-induced energy transfer and biexponential fluorescence lifetime analysis. The low-NA objective provides a larger field-of-view (FOV), thereby overcoming the limitations of a small FOV of the usually used high-NA objectives.

## Introduction

Cell adhesion and motility are important areas of research, which are closely linked to many key biological processes such as tumor cell invasion and metastasis, tissue formation and regeneration, and epithelial–mesenchymal transition^1–5^. With the rapid technological advances of lasers and fluorophores, many ingenious nanometer-sectioning optical imaging methods have been developed lately. TIRF microscopy^6–8^ is a standard imaging modality for membrane imaging in cell biology. Optical sectioning is acquired by using exponentially decaying evanescent waves within a few hundred nanometers from an interface. Two brilliant ideas have been introduced recently to enhance the axial resolution of TIRF microscopy: variable angle TIRF (vaTIRF) microscopy^9–11^, and differential evanescence nanometry (DiNa)^12^. vaTIRF uses the fact that the intensity distribution of an evanescence wave changes with the incident angle of an excitation laser beam. Nanometer-scale axial resolution is obtained by analyzing several TIRF images measured with different incident angles. In DiNa, two fluorescence images are acquired sequentially: one with total-internal reflection and the other with wide-field illumination. The axial positions of fluorophores are deduced from the intensity ratio of two images.

Supercritical-angle fluorescence (SAF) microscopy^13–16^ is a recently introduced nanometer-sectioning microscopy technique utilizing a tunneling effect of light emitted from fluorophores close to a water–glass interface. SAF is the transmission of light from a low refractive index medium (water) into a high refractive index medium (glass) when the transmitted angle is larger than the critical angle of a glass–water interface. It is a tunneling effect of photons, which can be observed only when light-emitting fluorophores are within a few hundred-nanometer distance from a water–glass interface. The axial positions of fluorophores are calculated by comparing transmitted light signals collected below and above the critical angle of the interface. The first SAF microscopy was built based on a scanning confocal microscope setup. A SAF imaging system based on TIRF microscopy was introduced later with direct optical nanoscopy with axially localized detection (DONALD)^16,17^. In DONALD, isotropic three-dimensional (3D) localization of molecules with 20 nm precision was demonstrated by combining direct stochastic optical reconstruction microscopy and SAF. Metal-induced energy transfer (MIET) imaging is another important microscopy technique capable of nanometer-scale axial localization of molecules by using fluorescence lifetime imaging microscopy (FLIM)^18–21^. It was discovered very early that a fluorophore’s excited-state lifetime can be shortened when there is a metal film close to it^22,23^. It is due to the resonant energy transfer from a fluorophore to a metal film, which results in fluorescence quenching. Since fluorescence lifetime changes sensitively with the distance between a fluorophore and a metal film, a FLIM image can be converted to a topographic map of fluorescence molecules with a nanometer-scale precision^24,25^. A confocal microscopy setup is employed to excite fluorophores within a short distance from a metal film. Various practical nanometer-sectioning MIET imaging systems and their applications have been demonstrated with 3–5 nm axial resolution and 100 nm measurement range^19–21^.

All of the above-mentioned nanometer-sectioning microscopy systems (vaTIRF, SAF, and MIET) use high-NA objectives to exclusively excite or collect signals from fluorophores within a few hundred-nanometer distance from an interface. A high-NA objective has a short focal length, which inevitably produces images with small FOVs. Therefore, the larger the NA of an objective, the higher the magnification. Small-FOV imaging is a severe disadvantage in many biomedical studies where cells’ statistical or cooperative behaviors need to be observed, e.g., cell-based high-throughput drug screening^26^, tumor cell invasion and metastasis^27^, and tissue formation and regeneration^3^. In this paper, we propose a novel imaging scheme that enables both nanometer-sectioning and large-FOV imaging possible. To convert measured lifetimes into nanometer-scale distances in MIET imaging, a critical constraint analogous to the one used in single-molecule localization microscopy should be satisfied ^28,29^; no more than one layer of excited fluorophores exists within the focal volume of an objective. A confocal microscope with a high-NA objective is used to satisfy this constraint in conventional MIET imaging. We used a low-magnification objective to obtain large-FOV images. Since most fluorescence lights radiated through a metal film into a glass substrate have large emission angles^20^, a high-NA objective is necessary when an inverted microscope setup is used in MIET imaging. In order to utilize a low-NA objective, we used an upright confocal setup. The constraint for single-molecule localization is satisfied by extracting shorter lifetime components with biexponential signal analysis. Since the lifetime shortening of fluorophores takes place within a few hundred-nanometer distance from a metal film in MIET, the constraint for single-molecule localization is automatically satisfied regardless of the NA of an objective if we use only the shortened lifetimes of biexponential lifetime signals.

## Results

**Fig. 1:**
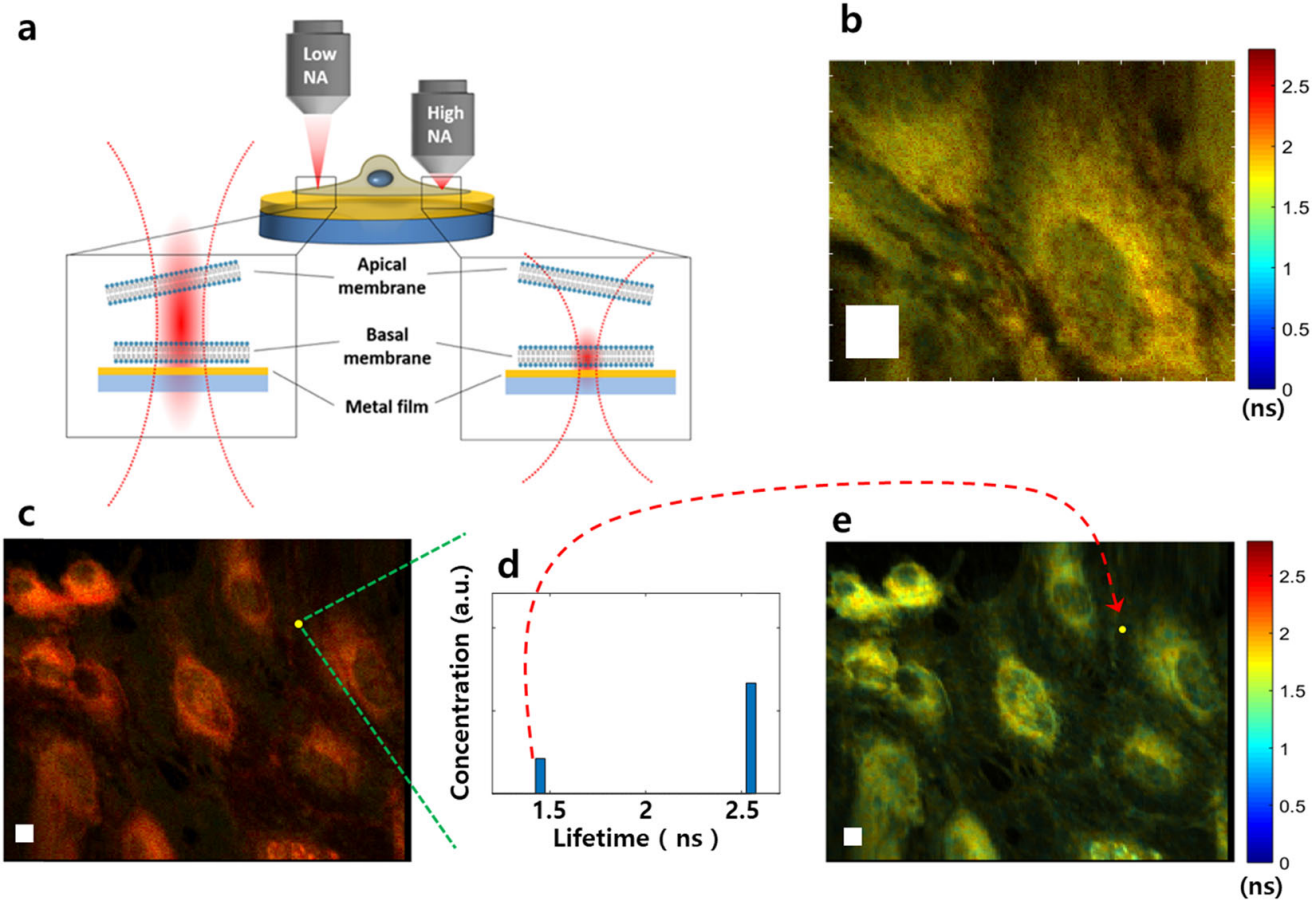
Large-FOV FLIM with biexponential signal analysis. **a** Geometry of apical/basal membranes of a cell, a metal film coated substrate, and the axial intensity profiles of excitation laser beams with high-NA and low-NA objectives. A high-NA objective is used in conventional MIET imaging to excite or collect signals from the basal membrane. **b** Small-FOV FLIM image measured with a high-NA objective (NA = 1.49, ×60) for human aortic endothelial cells (HAECs) stained with CellMask™ Green. HAECs are grown on a cover glass with a thin Au film on it. The image consists of 254 × 196 pixels, and its FOV is 40 μm × 32 μm. **c** Large-FOV FLIM image of the same HAEC sample measured with a low-NA objective (NA = 1.0, ×20). It has 254 by 196 pixels and covers a large-FOV of 120 μm × 96 μm. **d** Extracted lifetime components, and their concentrations for a specific pixel in (**c**). A fast deconvolution algorithm based on a library of exponentials (“Method”) is used. The longer lifetime is for the apical membrane of a cell, and the shorter one is for the basal membrane. **e** Reconstructed FLIM image from (**c**) by using the shorter lifetime of each pixel. White boxes are squares of 5 μm side length.

The feasibility of our proposed method is demonstrated by taking FLIM images of membrane-stained cells with high-NA and low-NA objectives (Fig. 1). Human aortic endothelial cells (HAECs) grown on a gold-coated coverslip were stained with CellMask™ Green (CMG) membrane stain and fixed for imaging. The peak emission wavelength of CMG is 530 nm with a single exponential decay lifetime of 2.43 ns (“Methods”, Supplementary Fig. 11). FLIM images are acquired using a custom-made laser-scanning confocal microscope (LSCM) and direct time-domain waveform measurements^30^ (“Methods”, Supplementary Fig. 1). Figure 1b shows a small-FOV FLIM image measured with a high-NA objective (×60, NA = 1.49). This objective is for TIRF microscopy and is not normally used in an upright microscope. We utilized it just for a comparative study. All images measured with the high-NA objective are obtained by conventional MIET imaging without biexponential signal analysis. Its FOV (40 μm × 32 μm) is not large enough to cover the entire shape of a cell. We set the pinhole size of the confocal system to 1.0 Airy unit to obtain a thin axial resolution of 0.62 μm (Supplementary Fig. 2). The axial position of the sample was carefully adjusted, such that the basal membranes of cells are placed at the focal plane of the imaging system (Supplementary Fig. 15). We obtained an average lifetime of 1.70 ns, which is much shorter than CMG’s original lifetime. All images in this report consist of 254 × 196 pixels.

Figure 1c is a large-FOV FLIM image of the same HAEC sample measured with a low-NA objective (×20, NA = 1.0). More than ten cells are within its FOV (120 μm × 96 μm), which is nine times larger than that of Fig. 1b. The pinhole size was set to 5.0 Airy unit to increase photon detection efficiency. The photon collection efficiency of the high-NA objective was three times larger than that of the low-NA objective. Due to this large pinhole size, we have a low axial resolution of 6.3 μm (Supplementary Fig. 2). The average lifetime of this image is 2.16 ns, which is much longer than that of the small-FOV FLIM image shown in Fig. 1b. A shortened lifetime is extracted from a biexponential decay signal for each pixel by using a library-function-based deconvolution algorithm (“Methods”). Figure 1d shows a typical example of two extracted lifetimes and their concentration ratio for a specific pixel in the large-FOV FLIM image. Once the shortened lifetimes are extracted, we reconstruct the original image by using the shortened lifetimes. Figure 1e is a new FLIM image reconstructed from Fig. 1c. The computation time of the reconstruction process for an image size of 254 × 196 pixels was about 6 min. The same process took several hours when we used a nonlinear least-squares algorithm. The average lifetime of Fig. 1e is 1.49 ns, which is even shorter than that of Fig. 1b (1.7 ns).

**Fig. 2:**
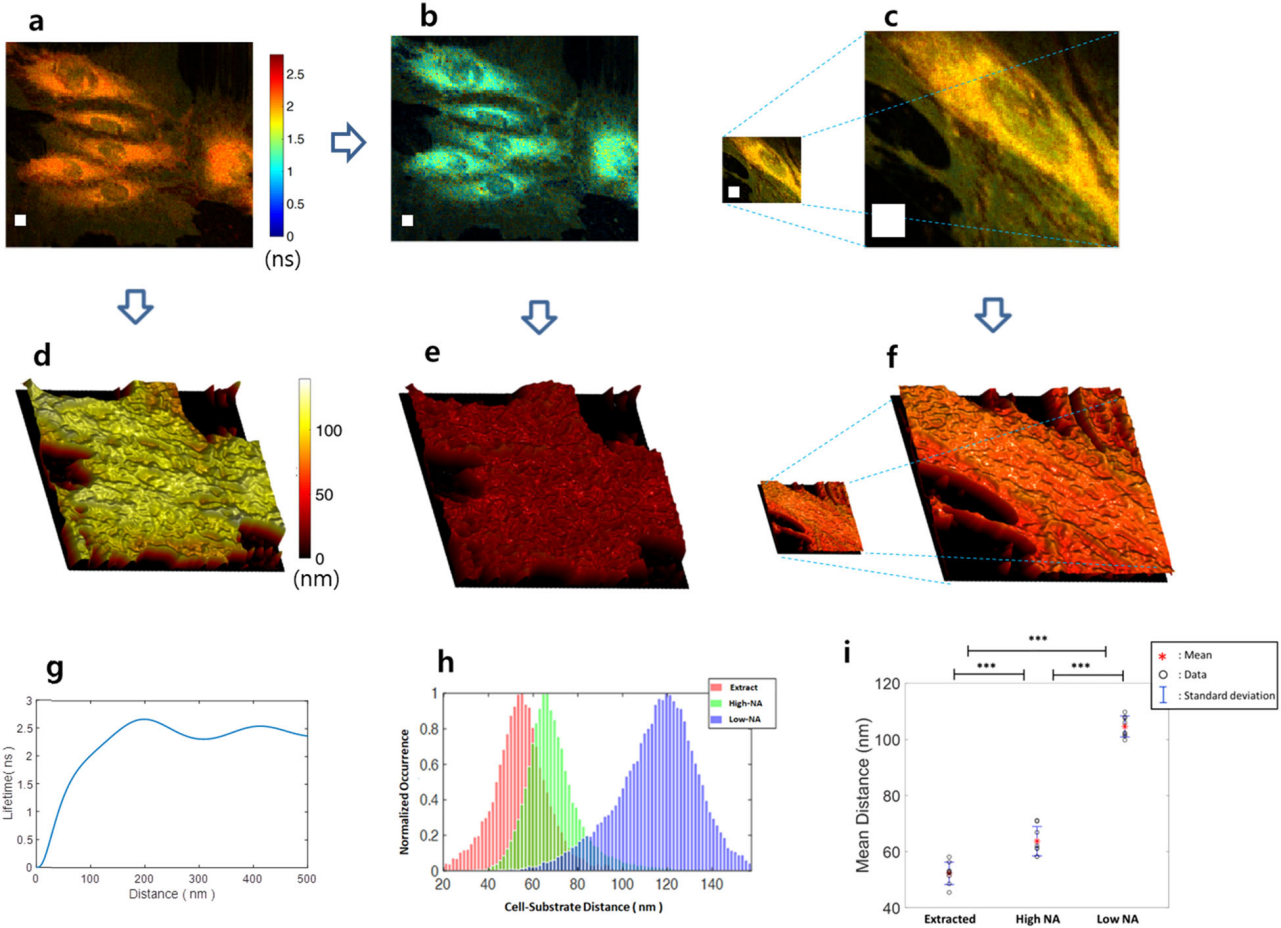
3D surface plots of cell–substrate distance measured with high-NA and low-NA objectives. **a** Large-FOV (120 μm × 96 μm) FLIM image of HAECs measured with the low-NA objective. **b** Reconstructed FLIM image with shorter lifetimes calculated by using biexponential signal analysis. **c** Small-FOV (40 μm × 32 μm) FLIM image measured with the high-NA objective for the HAECs sample. White boxes in (**a**–**c**) are squares of 5 μm side length. **d–f** 3D surface plots of cell–substrate distance converted from three FLIM images shown in (**a**–**c**). **g** Lifetime-to-distance conversion curve by the CPS model (“Methods”). **h** Normalized distributions of cell–substrate distances for the three images shown in (**d**–**f**). **i** Average cell–substrate distances and their standard deviations for the three surface profiles shown in (**d**–**f**). Eight different cell images were measured for statistical analysis.

FLIM images are converted into 3D topographic maps by using MIET (Fig. 2). Figure 2a, c are another pair of FLIM images for the same HAEC sample obtained with the low-NA and the high-NA objectives, respectively. Fluorescence signals in Fig. 2a are analyzed with a biexponential decay model (“Methods”). Figure 2b is a reconstructed FLIM image obtained from Fig. 2a using each pixel’s shorter lifetime component calculated from the biexponential signal analysis. We used a lifetime-to-distance (LTD) curve shown in Fig. 2g. The LTD curve was obtained with the well-accepted model of Chance, Prock, and Silbey (CPS model)^22^ (“Methods”) by measuring the optical properties of CellMask™ Green and the Au film of 51.6 nm thickness (Supplementary Figs. 11, 12)^18,19^. Due to an oscillatory behavior, the conversion range of the LTD curve is limited from 0 to 125 nm. The average slope of the LTD curve from 0 to 125 nm distance range is about 0.017 ns/nm. We transformed the three FLIM images shown in Fig. 2a–c into three topographic images of Fig. 2d–f by using the LTD curve.

For statistical analysis, we measured eight more pairs of large-FOV and small-FOV FLIM images and reconstructed another eight FLIM images by using the biexponential signal analysis (Supplementary Fig. 9). The same conversion procedures shown in Fig. 2 were followed to obtain three groups of eight images: high-NA, low-NA, and extracted-image groups (Supplementary Fig. 10). Figure 2h shows the three normalized histograms of the three different groups of images. The means and the standard deviations of measured cell–substrate distances for the three histograms are shown in Fig. 2i. The standard deviation of the conventional MIET imaging shown in the high-NA group is slightly smaller than that of our proposed method shown in the extracted group. Average cell–substrate distances for extracted, high-NA, and low-NA groups are 52, 63, and 104 nm, respectively (Fig. 2i).

We did biexponential signal analysis for the high-NA group. Results suggest that some fluorescence signals from apical membranes or internalized fluorophores are included in the high-NA group (Supplementary Fig. 7a). Results show that biexponential analysis can reduce the effect of epical membranes in cell-to-substrate distance measurement even for high-NA objective data (Supplementary Fig. 14). Another error source for the high-NA group is internalized fluorophores into a cell (Supplementary Fig. 13). Since the axial resolution of the confocal system for the high-NA group is 0.62 μm, any internalized fluorophores without MIET but located within this distance from the substrate make cell–substrate distances look larger than the actual distance. These two error sources make the high-NA group has a larger average cell–substrate distance than the extracted group. We used an upright confocal system for direct comparison of small- and large-FOV FLIM images with high- and low-NA objectives. Results in Fig. 2i show that the average cell–substrate distance of our proposed method is smaller than that of conventional MIET imaging.

The accuracy of extract lifetimes in the library-function-based deconvolution algorithm was tested with a Monte-Carlo simulation (Supplementary Fig. 8). Results show that the figure-of-merit (FOM) of our lifetime measurement method is about 10 when the shortened lifetime is *τ*_*b*_ = 1.5 ns (Supplementary Fig. 8c). The FOM of a single exponential decaying fluorophore was measured to be about 1.4 for the lifetime measurement system we used. The standard deviation of a measured lifetime becomes 0.047 ns when the number of photons used for measurement is 100,000. We can convert this lifetime error to a 2.78 nm cell-to-substrate distance error by using the LTD curve shown in Fig. 2g. When we used an excitation laser of 0.16 mW average power and an objective of NA = 1.0, it took about 2 min to collect 100,000 average number of photons per pixel for an image of 254 × 196 pixels. If we use a lower-NA objective, FOV can be further increased with the expense of less photon collection efficiency. Since accuracy is proportional to the number of collected photons in FLIM, this causes either longer measurement time or less measurement accuracy.

The FOV of an optical imaging system becomes smaller when we increase the resolution of the imaging system. Because of this complementary relation between FOV and optical resolution in microscopy, all lately developed nanometer-sectioning imaging methods produce small-FOV images. In this paper, we propose and demonstrate that this complementary relation can be broken by using MIET, and we can obtain nanometer-sectioning large-FOV optical images. Large-FOV images are obtained by using a confocal microscope with a magnification objective. Nanometer-scale axial sectioning is achieved by combing MIET and biexponential fluorescence signal analysis. Since our proposed method does not require a high-NA objective or the total-internal-reflection condition for thin axial sectioning illumination, there exists much flexibility in imaging system design. Our method can be used both with confocal and wide-field FLIM imaging systems. The feasibility of our proposed method was experimentally demonstrated with an upright confocal FLIM system for membrane-stained HAECs grown on an Au-coated glass substrate. Large FOV nanometer-sectioning topographic images of multiple cells are presented for cell-to-substrate distance measurement. We believe that our proposed method can open a new pathway toward large-FOV super-resolution microscopy and have a powerful impact on many cell biomedical research fields.

## Methods

### Custom-built confocal FLIM setup

We used a custom-made LSCM for taking fluorescence lifetime images of a sample (Supplementary Fig. 1). Fluorescence lifetimes were obtained with the analog-mean-delay method^30,31^, where fluorescence intensities are directly measured in the time-domain with a fast detector and a digitizer after exciting fluorophores with a picosecond laser. A gain-switched semiconductor laser (LDH-P-C-485, PicoQuant) producing 100 ps pulse width at 485 nm wavelength is used as an excitation laser source. The pulse repetition rate and the average power of the laser are 10 MHz and 160 μW, respectively. The laser output is delivered through a single-mode fiber to a custom-made upright confocal microscope. An optical bandpass filter is used to block spontaneous emission light from the laser. Two Galvano mirrors (X–Y Galvano scanner) are used to scan a focused laser beam on a sample. We used a high-NA objective (APON60XOTIRF, NA = 1.49, ×60, oil immersion, Olympus) for small-FOV imaging and a low-NA objective (XLUMPLFLN20XW, NA = 1.0, ×20, water immersion, Olympus) for large-FOV imaging. Emitted fluorescence light passes a dichroic mirror (DMLP505, Thorlabs) and an optical long-pass filter (FEL0500, Thorlabs) before it is coupled into a 50 μm-diameter multimode fiber (MMF). A confocal pinhole in front of the MMF is to control the axial resolution of the LSCM. For small-FOV imaging, we used a pinhole with a diameter of 1.0 Airy unit and obtained an axial resolution of 0.6 μm (Supplementary Figs. 2, 3). For large-FOV imaging, the pinhole size was set to 5.0 Airy unit, and the axial resolution was measured to be 6.4 μm. Fluorescence light is collected with a photomultiplier tube (R7400, Hamamatsu). High-frequency components of the detected signal were cut by a Gaussian low-pass filter, which has 60 dB voltage attenuation at 1.5 GHz frequency before they were sampled with a 12-bit digitizer with 3 GSa/s sampling-rate (EON-Express, Gage). The effective number of bits of the digitizer was measured to be 9.0.

We have measured 3D point spread functions (PSFs) for the low-NA and high-NA objectives used in our experiments. For the high-NA objective, we used a small confocal pinhole of 1.0 Airy unit (AU), which corresponds to 25.0 μm (Supplementary Fig. 3). For the low-NA objective, we used a large pinhole whose diameter is 5.0 AU, which is 60.2 μm. Fluorescent beads of 100 nm diameter (F8803, Thermo Fisher Scientific Inc.) are sparsely put in an agarose gel and used as point sources to measure PSFs. The peak wavelength of fluorescence light was 512 nm. A 3D PSF is measured by taking a series of 2D transverse images in the *x*–*y* plane while the sample was translated along the axial direction (*z*-axis) by a motorized stage. Axial resolution for the high-NA objective with 1 AU pinhole diameter was measured to be 0.62 μm, and that for the low-NA objective with 5 AU pinhole diameter was 6.4 μm (Supplementary Fig. 2). By comparing these results with theoretical curves^32^ (Supplementary Fig. 3), we estimate the effective NA of the high-NA and low-NA objectives are 1.2 and 0.85 instead of 1.49 and 1.0, respectively.

### Library-function-based deconvolution algorithm

We used a fast deconvolution algorithm based on a least-square curve-fitting method with a library of exponentials^33^. A model function with *N* lifetime components is defined as

1 $$h\left( t \right) \,=\, c_1b_1(t) \,+\, c_2b_2(t) \,+\, c_3b_3(t) \,+\, \cdots c_Nb_N(t),$$

where *b*_*k*_(t) = exp(−*t*/*τ*_*k*_); *k* = 1, 2 ⋯ *N* represents the library of exponential functions. {*τ*_1_, *τ*_2_, ⋯, *τ*_*N*_} are the equally spaced time constants of library functions. {*c*_1_, *c*_2_, ⋯ *c*_*N*_} are concentrations of library functions, which need to be solved by comparing the model function with a measured fluorescence intensity. When {*t*_1_, *t*_2_, ⋯, *t*_*M*_} is an equally spaced time sequence for the library functions, the model function *h(t)* can be represented by a column vector of equally spaced sampled data set *h* = (*h*_1_, *h*_2_, ⋯ *h*_*M*_)^*T*^ with *h*_l_ = *h*(*t*_*l*_); *l* = 1, 2⋯*M*. Similarly, we can represent a library functions *b*_*k*_(t) with a column vector *b*_*k*_ = (*b*_1*k*_, *b*_2*k*_, ⋯ *b*_*Mk*_)^*T*^ with *b*_*lk*_ = *b*_*k*_(*t*_*l*_). If we define an M-by-N matrix ≡ [*b*_1_, *b*_2_,⋯,*b*_*N*_], and a column vector *c* ≡ (*c*_1_, *c*_2_, ⋯ *c*_*N*_)^*T*^, Eq. (1) can be written as

2 $$h \,=\, Bc.$$

We measure the impulse response function of a fluorescence lifetime measurement system *IRF(t)* and define another column vector IRF by using the measured data. We define an M-by-M Toeplitz matrix *U* from a sampled column vector of IRF to represent the convolution integral between the *IRF(t)* and the model function *h(t)*: *Uh* = *UBc*. A library of exponential functions and their convolutions with the IRF are illustrated in Supplementary Fig. 5. When a measured time-domain fluorescence intensity data is given by a column vector *g* = (*g*_1_, *g*_2_, ⋯ *g*_*M*_)^*T*^, *g*_*l*_ = *g*(*t*_*l*_), we find the best nonnegative fitting parameters of {*c*_1_, *c*_2_, ⋯ *c*_*N*_} by using a nonnegative least-squares (NNLS) algorithm^34^ with a Tikhonov regularization term of:

3 $$\min \frac{1}{2}\parallel UBc \,-\, g\parallel ^2 \,+\, \frac{1}{2}\mathop {\sum}\nolimits_{n \,=\, 1}^N {(c_n)^2} .\quad (c_k \,\ge\, 0)$$

This NNLS algorithm can be used for any multiexponential decay signal. Here, we consider a simple case where a fluorophore has a single exponential decay component (CellMask™ Green) is used. When HAECs stained with CellMask™ Green are on a metal-coated substrate, a collected fluorescence signal by a low-NA objective can be written with the following biexponential decay function.

4 $$I(t) \,=\, \alpha \,\exp \left( { - \frac{t}{{\tau _r}}} \right) \,+\, \beta \,\exp \left( { - \frac{t}{{\tau _b}}} \right).$$

*τ*_*r*_ is a reference lifetime, which corresponds to the original fluorescence lifetime of a probe, *τ*_*b*_ represents a shortened fluorescence lifetime due to MIET.

To set the range of time constant for the library functions {*τ*_1_, *τ*_2_, ⋯ *τ*_*N*_}, we measured two different FLIM images of membrane-stained HAEC by using the high-NA objective. One FLIM image is for HAEC grown on a glass substrate with a metal film, and the other is without a metal film (Supplementary Fig. 4). Results show that *τ*_*r*_ is 2.43 ns, and *τ*_*b*_ is 1.70 ns. The standard deviation of the reference lifetime (*σ*_*r*_) was 0.064 ns. We set the upper bound lifetime *τ*_*N*_ of the library function to 2.6 ns (*τ*_*r*_ + 3*σ*_*r*_). The lower bound lifetime *τ*_1_ can be set by trial and error. If it is too small, the computational time becomes long, and the accuracy of results is degraded. If it is too large (close to *τ*_*r*_), we may not detect fluorophores close to a substrate. It is set to 0.5 ns in our case. An example of a measured fluorescence intensity signal and its deconvolution results are shown in Supplementary Fig. 6 for *N* = 85. More deconvolution results are shown in Supplementary Fig. 7. Only two or three components of 85 library functions have nonzero values. The shortened lifetime *τ*_*b*_ is calculated with the weighted mean lifetime of the model function given in Eq. (1). Among nonzero lifetime components calculated from our deconvolution algorithm, long lifetime components near *τ*_*r*_ = 2.43 ns are from the epical membranes of the sample. The effect of the apical membrane is removed by setting all the coefficients *c*_*n*_ to be zero for *τ*_*n*_ > (*τ*_*r*_ − 3*σ*_*r*_) = 2.24 ns.

The accuracy of the library-function-based deconvolution algorithm was tested for various measurement conditions by using numerically generated signals with a Monte-Carlo method (Supplementary Fig. 8). Results of Monte-Carlo simulations show that the FOM^31^ of our lifetime extraction method is about 10, and it increases abruptly as the difference of two lifetimes (*τ*_*r*_ − *τ*_*b*_) becomes smaller than 0.3. Results also show that the computational speed of our method is much faster than that of the nonlinear least-squares method regardless of the difference of two lifetimes (Supplementary Fig. 8d).

### Lifetime-to-distance conversion

In order to convert FLIM images to topographic maps, we used the well-known CPS model, which was first introduced in 1978 by Chance, Prock, and Silbey^22,35,36^, and experimentally verified later^5,37^. Spontaneous emission of light from a fluorescence molecule can be described by a simple damped harmonic oscillator model for a radiating dipole.

5 $$\frac{{d^2p}}{{dt^2}} \,+\, b_0\frac{{dp}}{{dt}} \,+\, \omega _0^2p \,=\, 0.$$

Here *p* is the electric dipole moment, *b*_0_ is the damping constant, *ω*_0_ is the resonant angular frequency of the dipole without a damping term, m is the effective mass, and e is the effective charge of a dipole. If we assume a solution as *p* = *p*_0_ exp(−*iΩt*) with *Ω* = *ω* − *ib*/2, we have

6 $$\omega ^2 \,=\, \omega _0^2 \,-\, \frac{{b_0^2}}{4},\quad b \,=\, b_0.$$

*τ*_0_ = 1/*b*_0_ is the lifetime of a damped oscillating dipole in free space. When there is a thin metal film near an emitter, the equation of motion for a dipole can be modeled by a forced damped harmonic oscillator with

7 $$\frac{{d^2p}}{{dt^2}} \,+\, b_0\frac{{dp}}{{dt}} \,+\, \omega _0^2p \,=\, \frac{{e^2}}{m}E_R,$$

where *E*_*R*_ is reflected electric field at the dipole position by the metal film. For a given reflected field *E*_*R*_ ≡ *E*_0_ exp(−*iΩt*), we can assume a solution for Eq. (7) as *p* = *p*_0_ exp(−*iΩt*) with *Ω* = *ω* − *ib*/2. Then, have

8 $$\omega ^2 \,=\, \omega _0^2 \,+\, \frac{{b^2}}{4} \,-\, \frac{{bb_0}}{2} \,-\, \frac{{e^2}}{{mp_0}}{\it{Re}}\left\{ {E_0} \right\},\quad \frac{b}{{b_0}} \,=\, 1 \,+\, \frac{{e^2}}{{mp_0\omega b_0}}{\it{Im}}\left\{ {E_0} \right\},$$

where *ω* is the modified angular frequency of a dipole, and *b* is the modified damping constant. Both *ω* and *b* are modified by the reflected electric field *E*_*R*_(*t*) from the metal film. To represent the phase delay of the reflected field *E*_*R*_(*t*) with respect to the oscillating dipole *p*(*t*), *E*_0_ must be a complex number. The phase and the amplitude of *E*_0_ are determined by the optical susceptibility of the metal film and the distance of the oscillating dipole from it. Once we obtain the reflected electric field *E*_*R*_(*t*) = *E*_0_ exp(−*iΩt*), the modified lifetime of the damped oscillating dipole by the metal film *τ* = 1/*b* can be calculated from Eq. (8). Since the angular radiation intensity patterns from an oscillating dipole are different for a perpendicular (⊥) and a parallel (||) dipole components, we calculate the reflected electric field *E*_*R*_(*t*) = *E*_0_ exp(−*iΩt*) for the two polarization directions. We expanded the radiating electric field from a dipole with a summation of plane waves and calculated the Fresnel’s reflection coefficients of each plane wave component for a three-layer structure: a thin Au film sandwiched by water and cover glass made of BK7 glass. When *z* is the distance from the radiating dipole to the metal film, the relative damping constant of each dipole polarization direction can be expressed as Eq. (9) and Eq. (10)^36^.

9 $$\frac{{b_ \bot }}{{b_0}} \,=\, 1 \,-\, QY \cdot \left( {1 \,-\, \frac{3}{2}{\it{Re}}\left\{ {{\int}_0^\infty {du} \frac{{u^3}}{{\sqrt {1 \,-\, u^2} }}\left[ {1 \,+\, R_p\exp \left( {2ik_{z1}z} \right)} \right]} \right\}} \right),$$

10 $$\frac{{b_\parallel }}{{b_0}} \,=\, 1 \,-\, QY \cdot \left( {1 \,-\, \frac{3}{4}Re\left\{ {{\int}_0^\infty {du} \frac{u}{{\sqrt {1 \,-\, u^2} }}\left[ {\left( {1 \,-\, u^2} \right)\left\{ {1 \,-\, R_p\exp \left( {2ik_{z1}z} \right)} \right\} \,+\, \left\{ {1 \,+\, R_s\exp \left( {2ik_{z1}z} \right)} \right\}} \right]} \right\}} \right).$$

*QY* stans for the quantum yield of a fluorophore. Since we could not find the *QY* of the CellMask™ Green (CMG) in literature, we have calculated it from the known *QY* of FITC (0.93)^38^. By using the absorption and the emission curves of FITC and CMG in DPBS (Supplementary Fig. 11), we obtained *QY* = 0.74 for CMG embedded into the membrane of a HAEC (Supplementary Note 1).

*b*_┴_ and *b*_‖_ are the damping constants of the vertical and the horizontal dipoles, respectively. *k*_1_(=*n*_1_*k*_0_) is the wave vector of a plane wave in a medium “1”. *k*_*t*1_ is the transverse component (projected on the *x*–*y* plane) of *k*_1_ with respect to the interface, and *k*_*z*1_ is the vertical component (along the *z*-axis) of *k*_1_ in the medium “1”. We have $k_1^2 \,=\, k_{t1}^2 \,+\, k_{z1}^2$. If we define *u* as the normalized transverse wave vector such as *u* ≡ *k*_*t*1_/*k*_1_, we have $k_{z1} \,=\, k_1\sqrt {1 \,-\, u^2}$. *R*_*p*,*s*_ denotes the reflectance of p-polarized or s-polarized light by a metal film (medium m) sandwiched by the upper-medium “1” and the lower-medium “2”. Then, we have

11 $$R_{p,s} \,=\, r_{p,s}^{1m} \,+\, \frac{{t_{p,s}^{1m}t_{p,s}^{m1}r_{p,s}^{m2}\exp (2ik_{zm}d)}}{{1 \,-\, r_{p,s}^{m1}r_{p,s}^{m2}\exp (2ik_{zm}d)}},$$

where $r_{p,s}^{ij}$ and $t_{p,s}^{ij}$ are the Fresnel’s reflection and transmission coefficients on an interface for s- and p-polarized lights from a medium “i” to a medium “j”, and *d* is the thickness of the metal film. We have $k_{zm} \,=\, k_0\sqrt {n_m \,-\, n_1u^2}$, where *n*_*m*_ is a complex-valued refractive index of the metal film.

The medium “1” is water, and we have *n*_1_ = 1.33 at 530 nm wavelength. The complex refractive index *n*_*m*_ and the thickness *d* of the Au film in Eq. (11) are measured by a spectroscopic ellipsometer, and we obtained *d* = 51.6 nm, and *n*_*m*_ = 0.23 + 2.22*i* at *λ*_*em*_ = 530 nm, the peak emission wavelength of CMG (Supplementary Fig. 11). The medium “2” is BK7 glass, and we used *n*_2_ = 1.52 at 530 nm wavelength^39^.

It was reported early that the orientation of CellMask Deep Red plasma membrane stain is random within cell membrane^18^. Since we used CellMask™ Green plasma membrane stain whose structure is very close to that of CellMask™ Deep Red, we assumed that its orientation is also isotropic. If it is not isotropic, we need to measure the percentage of fluorophores aligned to the vertical and horizontal axes and recalculate the LTD curve by using Eq. (12). When the orientation of a fluorophore is isotropic, the expectation value for damping constant *b* and fluorescence lifetime *τ* become

12 $$\frac{{\tau _0}}{\tau } \,=\, \frac{b}{{b_0}} \,=\, \frac{1}{3}\frac{{b_ \bot }}{{b_0}} \,+\, \frac{2}{3}\frac{{b_\parallel }}{{b_0}}.$$

The lifetime of CMG without a metal film was measured to be *τ*_0_ = 2.43 ns (Supplementary Fig. 4). By changing *z* from 0 to 500 nm, we calculated (*b*_┴_/*b*_0_) and (*b*_‖_/*b*_0_) as a function of *z* from Eq. (9) and Eq. (10). Then, we obtain *τ(z)* from Eq. (12) to calculate the LTD conversion curve shown in Fig. 2g. The average slope of the LTD curve from 0 to 125 nm distance range is about 0.017 ns/nm.

### Sample preparation

Sample cells were grown on top of a fibronectin-treated Au film and were fixed before imaging to maximize photon collection efficiency. We have used the following procedures to culture, stain, and fix samples. HAECs purchased from ATCC (Manassas, VA, USA) were the sample cells we have prepared. CellMask™ Green plasma membrane stain (Life Technologies, Carlsbad, CA, USA) was used to label the membranes of HAECs. Cells were cultured in Vascular Cell Basal Medium (ATCC, Manassas, VA, USA) supplemented with Endothelial Cell Growth Kit-VEGF (ATCC, Manassas, VA, USA) and Gentamicin-Amphotericin B Solution (Thermo Fisher Scientific, Waltham, MA, USA) at 37 °C in a humidified 5% CO_2_ incubator. We used a Fibronectin-treated (1%) gold-coated glass coverslip (16 mm in diameter). The average number of cells per well was about 2 × 10^4^. Another sample of HAECs was cultured on a regular coverslip without a metal film on it for a control experiment without the MIET effect. We used fibronectin from human plasma (Sigma-Aldrich, St. Louis, MO, USA). After 24 h, cells were stained with 1.5X CellMask™ Green plasma membrane stain for 10 min and fixed with 4% paraformaldehyde. Fixed cells were mounted using VECTASHIELD^®^ mounting medium (Burlingame, CA, USA) for imaging.

### Statistics and reproducibility

Anova one way test was used to calculate the *P* value. *P* values <0.001 was represented by three asterisks.

### Reporting summary

Further information on research design is available in the Nature Research Reporting Summary linked to this article.

## Supplementary information

Supplementary Information

Description of Additional Supplementary Files

Supplementary Data 1

Reporting Summary

## Data Availability

The data corresponding to Fig. 2g, h, i is shown in Supplementary Data 1. All other data that support the findings of this study is available from the corresponding author upon reasonable request.

## Code availability

We used matlab program to implement Library-function-based deconvolution algorithm. The matlab code is available from corresponding author upon reasonable request.
