# Supplementary material for: Large field-of-view nanometer-sectioning microscopy by using metal-induced energy transfer and biexponential lifetime analysis
Source: Commun Biol. 2021 Jan 19;4:91. doi: 10.1038/s42003-020-01628-3 (PMC7815909; doi:10.1038/s42003-020-01628-3)
Supplement: Supplementary file 1 — Supplementary Information [file 42003_2020_1628_MOESM1_ESM.pdf]

Supplementary information

Large field-of-view nanometer-sectioning microscopy by using metal-induced energy transfer and biexponential lifetime analysis

Wonsang Hwang[1], Jinwon Seo[2], DongEun Kim[1], Chang Jun Lee[1], In-Hong Choi[2], Kyung-Hwa Yoo[1], Dug Young Kim[1*]

[1]Dept. of Physics, Yonsei Univ., Seoul, Rep. of Korea,
[2]Dept. of Microbiology & institute for Immunology and Immunological Diseases, College of Medicine, Yonsei University, Seoul, Rep. of Korea,

Corresponding author: dykim1@yonsei.ac.kr

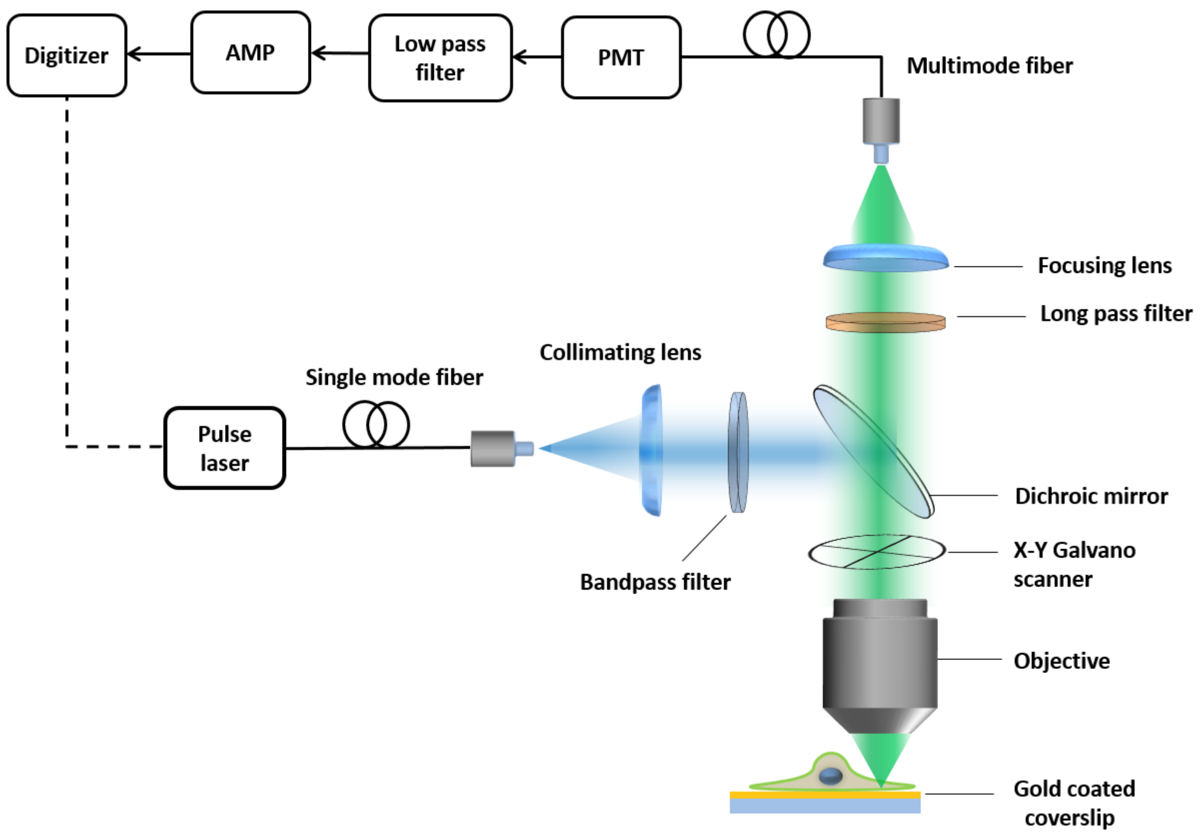

Supplementary Figure 1

Custom-built confocal fluorescence lifetime imaging microscopy (FLIM) setup

Pulse laser (LDH-P-C-485, PicoQuant) 100 ps pulse width, 10 MHz repetition rate, 160 μW average power, 485 nm peak wavelength; PMT (R7400, Hamamatsu), photomultiplier tube; AMP, Electronic amplifier; Low pass filter, electronic Gaussian low-pass filter; Digitizer (EON-Express, Gage), 3 GSa/s sampling-rate; Dashed line, sync signal from the pulsed laser to the digitizer.

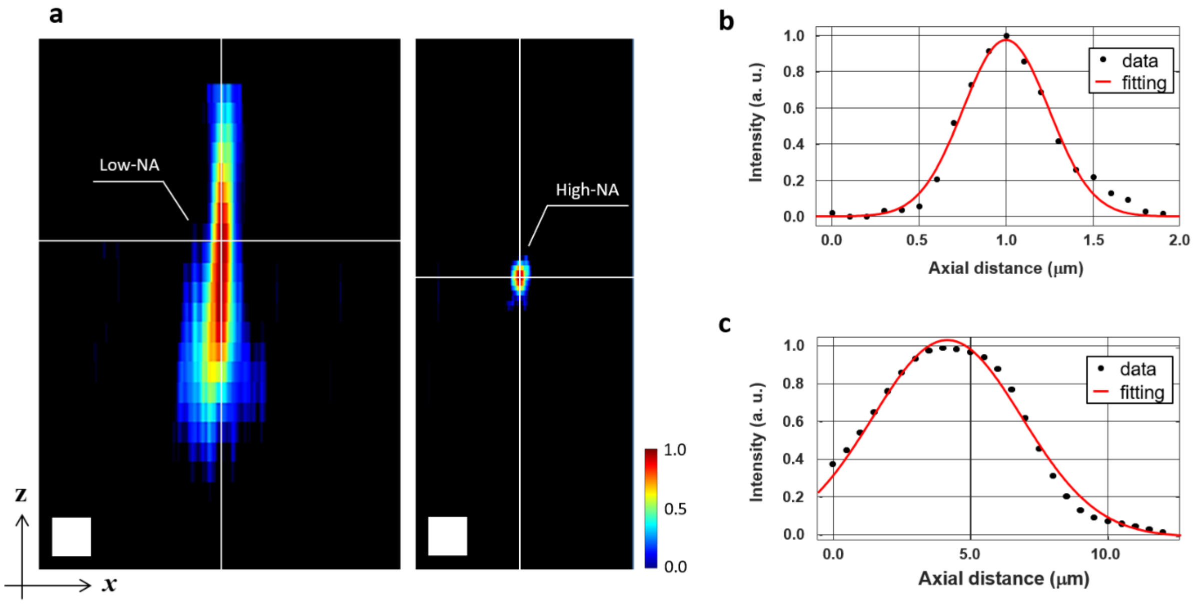

Supplementary Figure 2

Measured axial point spread functions (PSFs) for high-NA and low-NA objectives

(a) 2-D cross-sectional views of the two measured 3-D PSFs for the low-NA (left) and high-NA (right) objectives. The size of a pinhole in confocal microscopy is given with Airy unit (AU). One AU corresponds to (1.22 · λ / NA) · M, where λ is the average wavelength of excitation and emission wavelength, NA and M are the numerical aperture and the magnification of an objective[1]. For the high-NA objective, we used a small confocal pinhole of 1.0 Airy unit (AU), which corresponds to 25.0 μm. For the low-NA objective, we used a large pinhole whose diameter is 5.0 AU, which is 60.2 μm. The vertical axis of the figure (z-axis) is the axial direction, and the horizontal axis (x-axis) is one of the two transverse axes of a laser beam. Another transverse axis (y-axis) is fixed, such that the 2-D cross-sectional images cut through the peak intensity points of the 3-D PSFs. The pixel pitch along the axial direction (z-axis) is 500 nm for the low-NA objective and 100 nm for the high-NA objective. The pixel pitch along the transverse direction (x-axis) is 20 nm for both cases. The white boxes in each image is a square of 1 μm side-length. **(b)** 1-D intensity profile along the vertical white line shown in the 2-D cross-sectional image for the high-NA objective (a-right). The axial resolution defined as the full width at half maximum (FWHM) of this curve is 0.62 μm. **(c)** 1-D intensity profile along the vertical white line shown in the 2-D cross-sectional intensity image for the low-NA objective (a-left). The axial resolution is 6.4 μm. The transverse resolutions for the low-NA and high-NA objectives calculated along the horizontal white lines shown in (a) are 0.37 μm and 0.26 μm, respectively. Since the transverse resolution of an objective is given as (0.61 · λ / NA), we estimate the effective NA of the high-NA and low-NA objectives are 1.2 and 0.85, respectively.

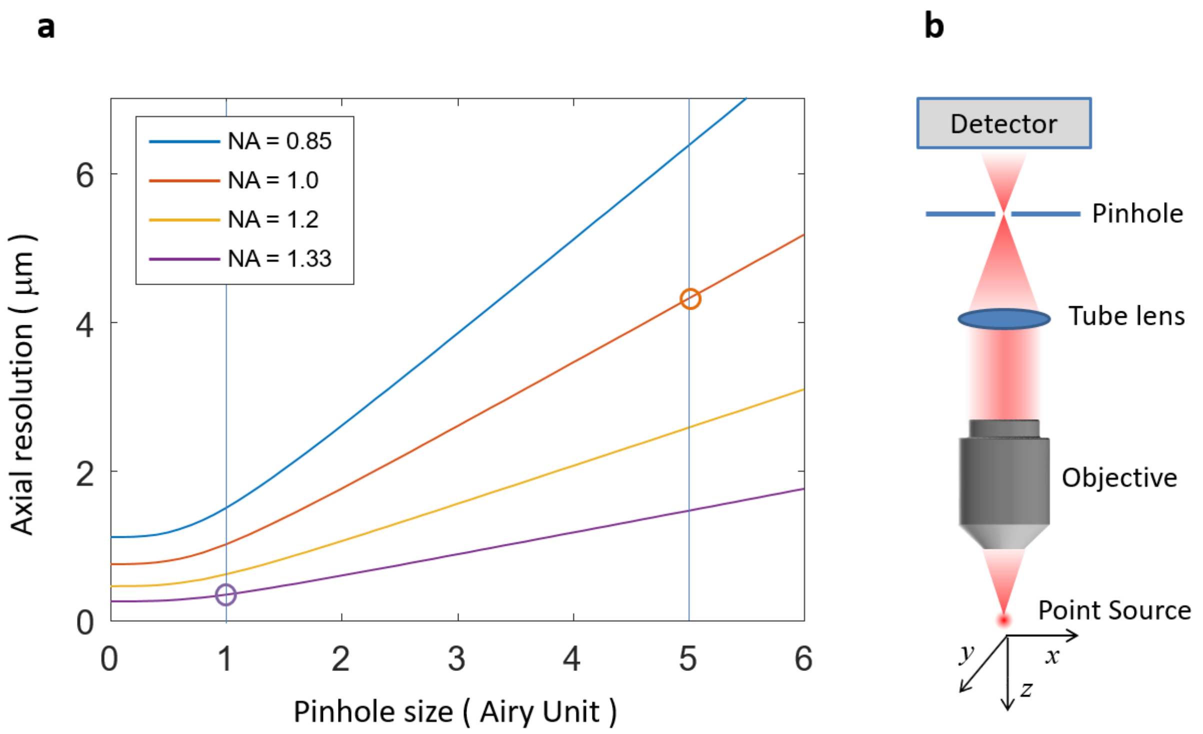

Supplementary Figure 3

Axial resolution v.s. pinhole size for various objectives in confocal microscopy

(a) Calculated axial resolution as a function of a pinhole size (diameter) for various objectives used in confocal microscopy. We used the cubic equation for the axial resolution calculation described in Ref[1], which is

$$\text{Axial resolution } = 0.67\lambda \cdot \sqrt[3]{1 + 1.47AU^3}/\left(n - \sqrt{n^2 - NA^2}\right).$$

The axial resolution is the full-width at half maximum of a detected signal as a function of a point source's axial position along the optical axis in the object space. λ is the wavelength of a laser, n is the refractive index of a sample medium, and AU is the pinhole size in Airy units. The diameter of a pinhole with 1 AU corresponds to $1.22\lambda \cdot M/$NA, where M is the magnification of an objective. We used $\lambda = 512$ nm, and n = 1.33. **(b)** A schematic diagram for the axial resolution measurement setup showing the position of a pinhole and a point source in confocal microscopy.

A small purple circle in (a) shows that the calculated axial resolution for a NA = 1.33 objective with a 1 AU diameter pinhole is 349 nm. A tiny red circle in (a) indicates that the axial resolution is 4.32 μm for a NA = 1.0 objective when the pinhole size is 5 AU. Measurement results of supplementary Fig. 2 show that axial resolution for the high-NA objective with 1 AU pinhole diameter is 0.62 μm. The axial resolution for the low-NA objective with 5 AU pinhole diameter is 6.4 μm. By comparing these experimental results with the theoretical curves in (a), we can deduce that the effective NA of the high-NA and low-NA objectives are 1.2 and 0.85 instead of 1.49 and 1.0, respectively. This is due to higher-order aberrations and refractive index mismatches in our experiments.

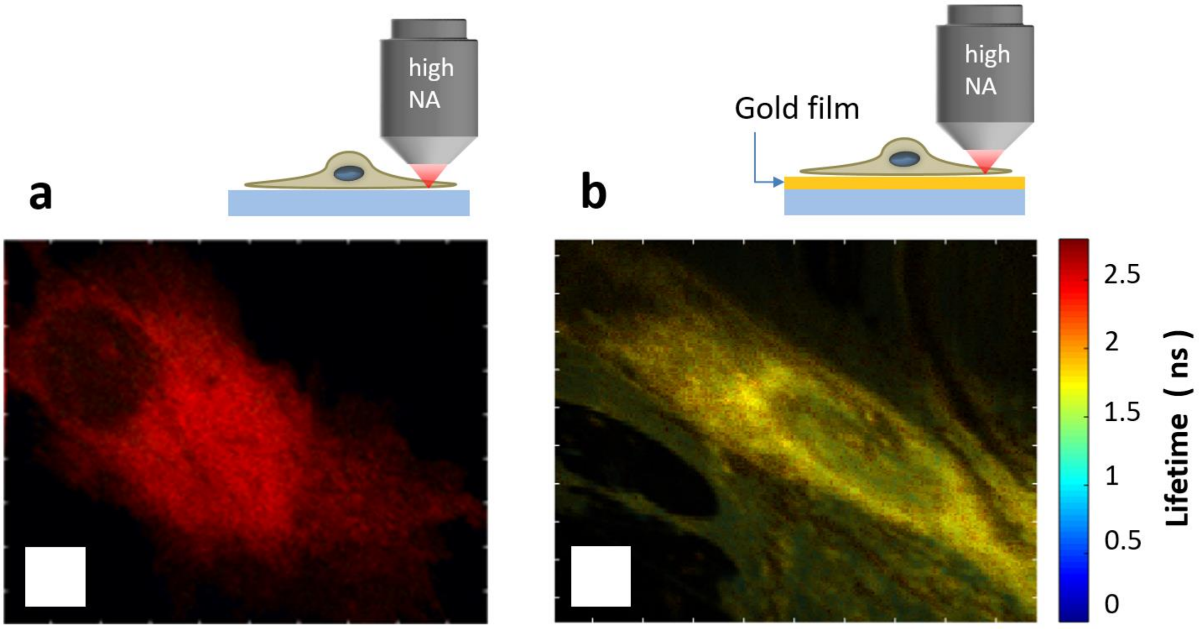

Supplementary Figure 4

Side-by-side comparison of FLIM images of a HAEC with and without a gold film

(a) A FLIM image of a human aortic endothelial cell (HAEC) cultured on a coverslip without a metal film. It is measured with the high-NA objective of NA = 1.49. The mean fluorescence lifetime of non-zero fluorescence intensity pixels is 2.43 ns. Image pixels whose fluorescence intensities are higher than three times the imaging system's noise level are counted. **(b)** Another FLIM image of a HAEC cultured on a coverslip with a thin gold film. It is also measured with the high-NA objective. The mean fluorescence lifetime is shortened to 1.70 ns due to MIET. Because of the thin axial resolution of the high-NA objective (0.62 μm), the samples' axial positions were carefully adjusted to make the basal membranes of cells located at the focal plane of the confocal microscope. Small white boxes in the images are squares of 5 μm side-length.

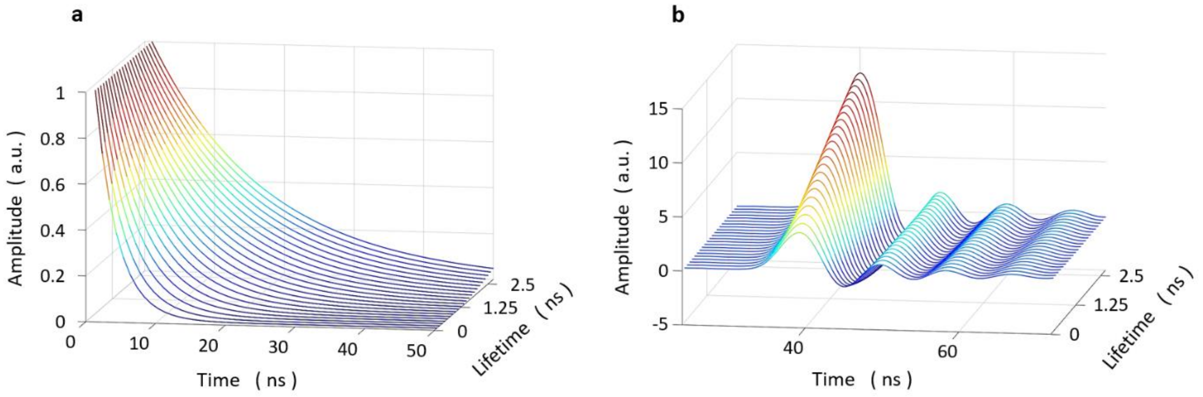

Supplementary Figure 5

A library of exponentials and its convolution with the impulse response function (IRF) of a measurement system

(a) A library of *N* exponential functions ($b_k(\mathrm{t}) = \exp(-t/\tau_k); k = 1, 2 \cdots N$) plotted from t = 0 to t = 50 ns. Lifetimes $\{\tau_1, \tau_2, \tau_3, \cdots, \tau_N\}$ are equally spaced from τ_1 = 0.5 ns to τ_N = 2.6 ns with *N* = 22. **(b)** Convolutions of library functions and the IRF of our measurement system. Each function is obtained by calculating the convolution of one of the exponential decay functions shown in (a) with the impulse response function of our experimental measurement system. Ringing in each plot is due to the parasitics of homemade analog circuits used in our measurement system.

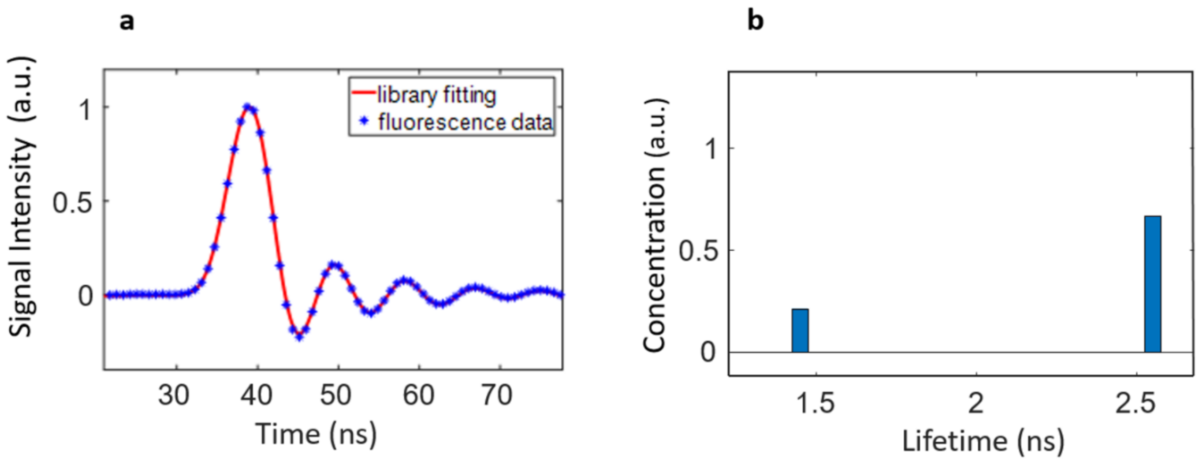

Supplementary Figure 6

Measured fluorescence intensity signal and its deconvolution results

(a) An example of measured fluorescence signal data and the best fitting curve calculated with our deconvolution method. 85 library functions (N = 85) were used with equally spaced lifetime constants from τ_1 = 0.5 ns to τ_N = 2.6 ns. **(b)** Concentrations of lifetime components calculated from the graph shown in (a).

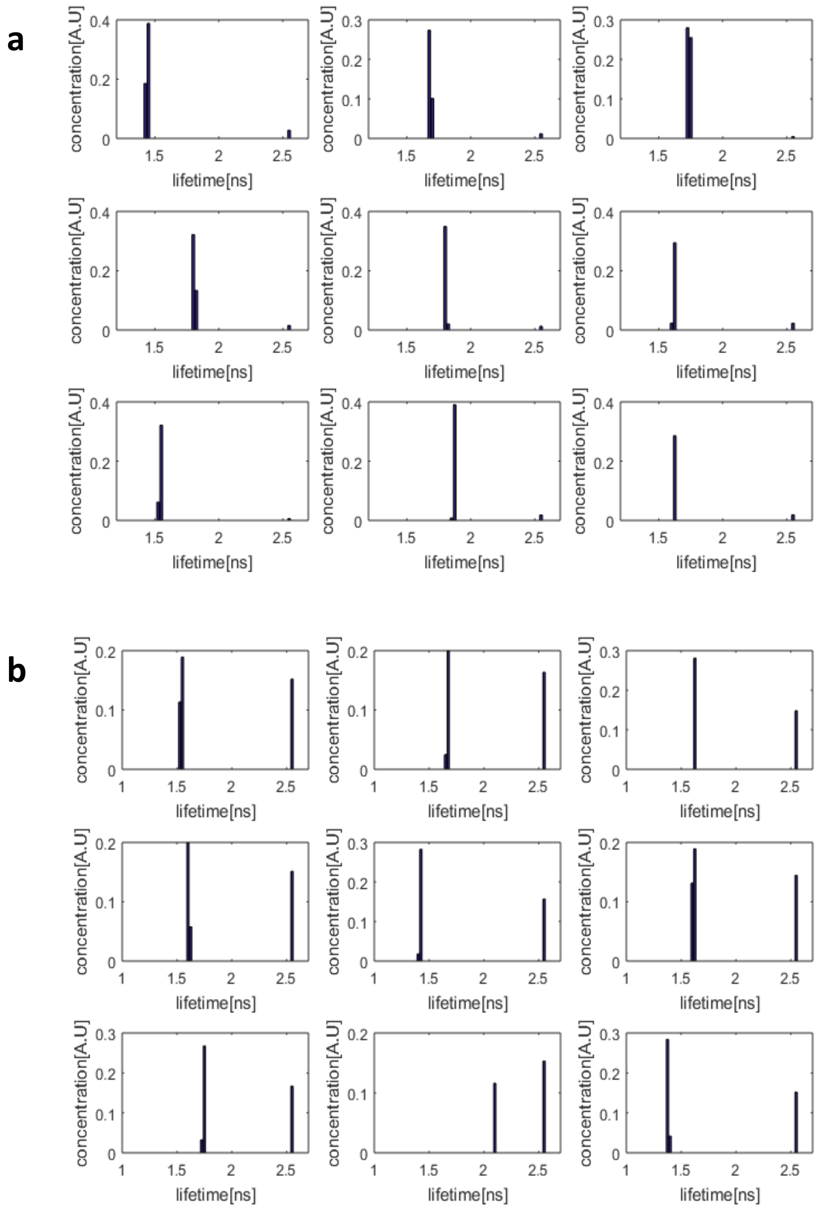

Supplementary Figure 7

Deconvolution results for fluorescence signals measured with high-NA and low-NA objectives

(a) Examples of deconvolution results of concentrations and lifetimes for measured fluorescence signals with the high-NA objective. **(b)** Representative deconvolution results of concentrations and lifetimes for measured fluorescence signals with the low-NA objective. 85 library functions (N = 85) were used with equally spaced lifetime constants from τ_1 = 0.5 ns to τ_N = 2.6 ns.

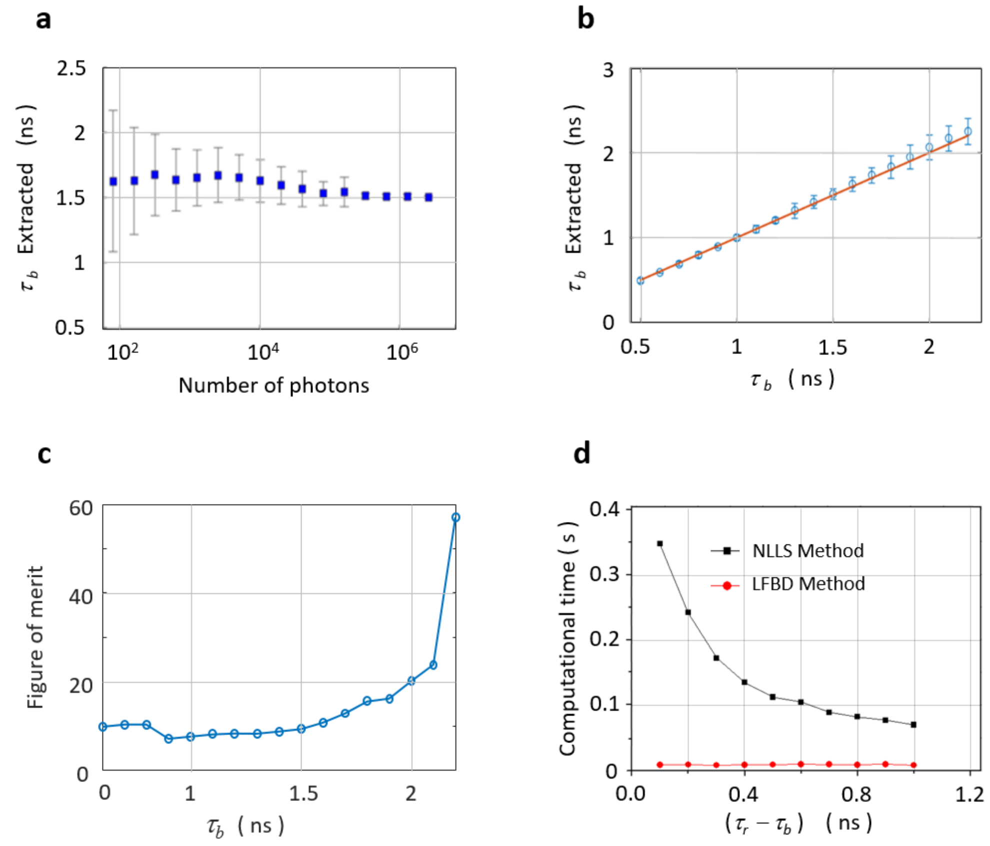

Supplementary Figure 8

Monte Carlo simulation for the accuracy test of the library-function-based deconvolution (LFBD) method

The accuracy and the photon economy of the LFBD method are tested with fluorescence signals numerically generated by a Monte Carlo method. We used 100 generated fluorescence intensity waveforms to calculate the mean and the standard deviation of one data point. We have added sampling noise in addition to the shot-noise of photons for the 3 GHz digitizer we have used in experiments. The effective number of bits of the digitizer was measured to be 9.0, and we used it for adding digitizing errors to numerically generated data. Shortened lifetime $\boldsymbol{\tau_b}$ is extracted from generated FLIM data by using the LFBD method with $\boldsymbol{\tau_1 = 0.5\ \mathrm{ns}}$, $\boldsymbol{\tau_N = 2.6\ \mathrm{ns}}$, *N = 85* (Methods). **(a)** Mean value of extracted lifetime $\boldsymbol{\tau_b}$ and its standard deviation ($\boldsymbol{\pm\sigma}$) as a function of the number of photons used for measurement. Fluorescence intensity signals were generated for $\boldsymbol{\tau_r = 2.43\ \mathrm{ns}}$, $\boldsymbol{\tau_b = 1.5\ \mathrm{ns}}$, and $\boldsymbol{\alpha = \beta = 0.5}$ in eq.(4) (Methods). **(b)** Mean value of extracted lifetime ($\boldsymbol{\tau_b}$ Extracted) and its standard deviation $\boldsymbol{\sigma}$ as a function of the actual lifetime $\boldsymbol{\tau_b}$. The number of photons used for generating each waveform was 100,000. **(c)** The figure of merit (FOM) of our lifetime measurement method as a function of the shortened lifetime $\boldsymbol{\tau_b}$ calculated from data shown in (b). The figure of merit (FOM)[2] of a FLIM system is defined as $(\boldsymbol{\sigma_\tau \cdot \sqrt{NoP}\ /\tau_b})$, where $\boldsymbol{NoP}$ is the number of photons used in measurement, $\boldsymbol{\sigma_\tau}$ is the standard deviation of measured lifetime, and $\boldsymbol{\tau_b}$ is the actual lifetime. A shot-noise-limited ideal FLIM system has FOM = 1. It shows that the FOM becomes worse as the shortened lifetime $\boldsymbol{\tau_b}$ is increased closer to the reference lifetime $\boldsymbol{\tau_r}$. **(d)** Side-by-side comparison of computational time to calculate a shortened lifetime $\boldsymbol{\tau_b}$ from a given biexponential decay signal for the nonlinear least-squares (NLLS) method and the LFBD method. As the lifetime difference between the reference and the shortened lifetimes ($\boldsymbol{\tau_r - \tau_b}$) is decreased, the computational time of NLLS method is increased much, while that of our LFDB method remains almost constant.

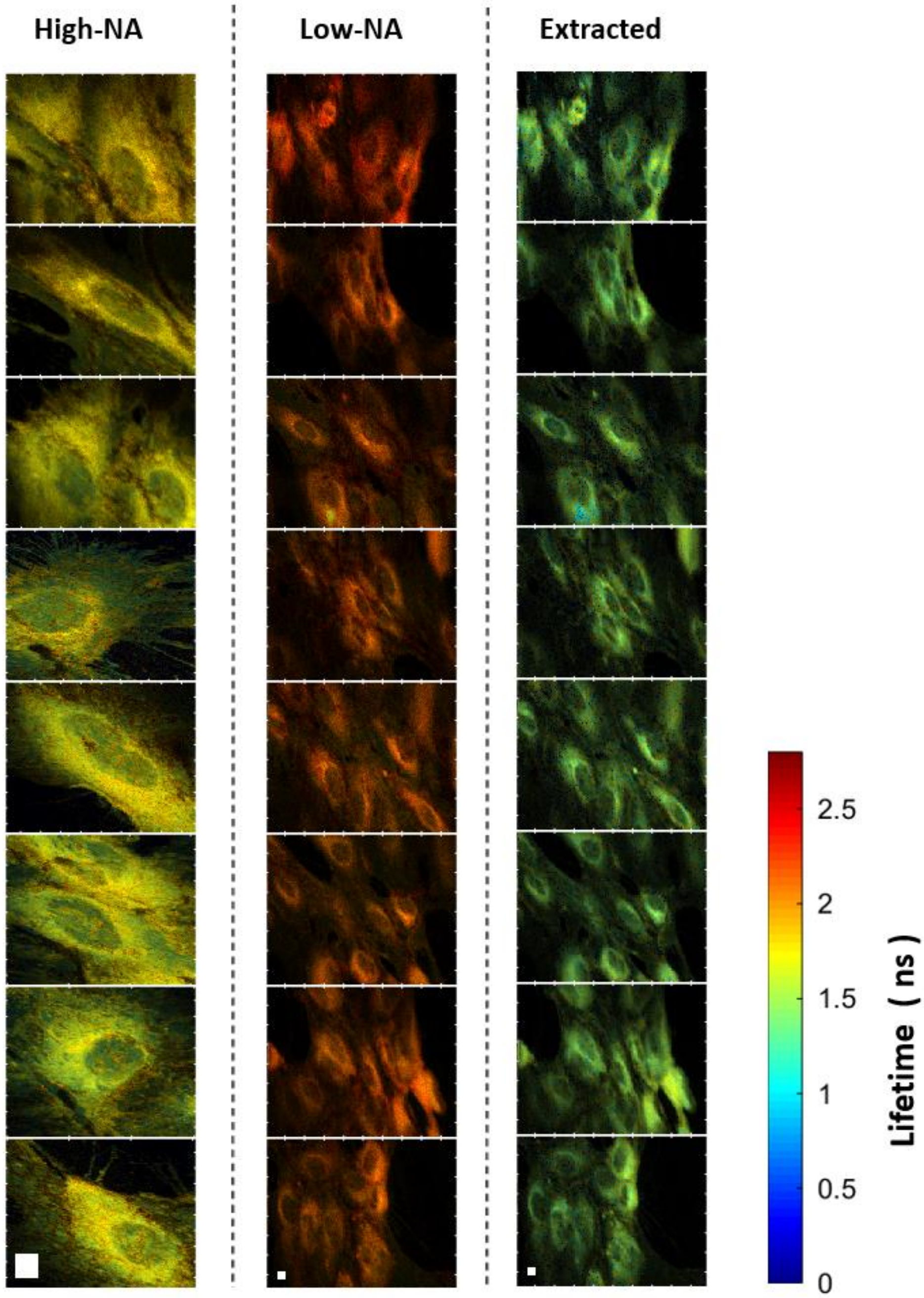

Supplementary Figure 9

Side-by-side comparison of high-NA, low-NA, and extracted FLIM images

FLIM images of human aortic endothelial cells (HAECs) on a thin metal film measured by three different methods: FLIM images using the high-NA objective, the low-NA objective, and our deconvolution method. Images in the left column are measured by the high-NA objective and have only one or two cells within a field of view. The mean lifetime of these images is 1.7 ns. Images in the central column are measured by the low-NA objective and have large FOVs. Their mean lifetime is 2.2 ns. Images shown in the right column are reconstructed from the images of the central column by using our proposed biexponential signal analysis method. These images' average lifetime is about 1.5 ns, which is the shortest among the three groups. Each white box in the image is a square of 5 μm side-length.

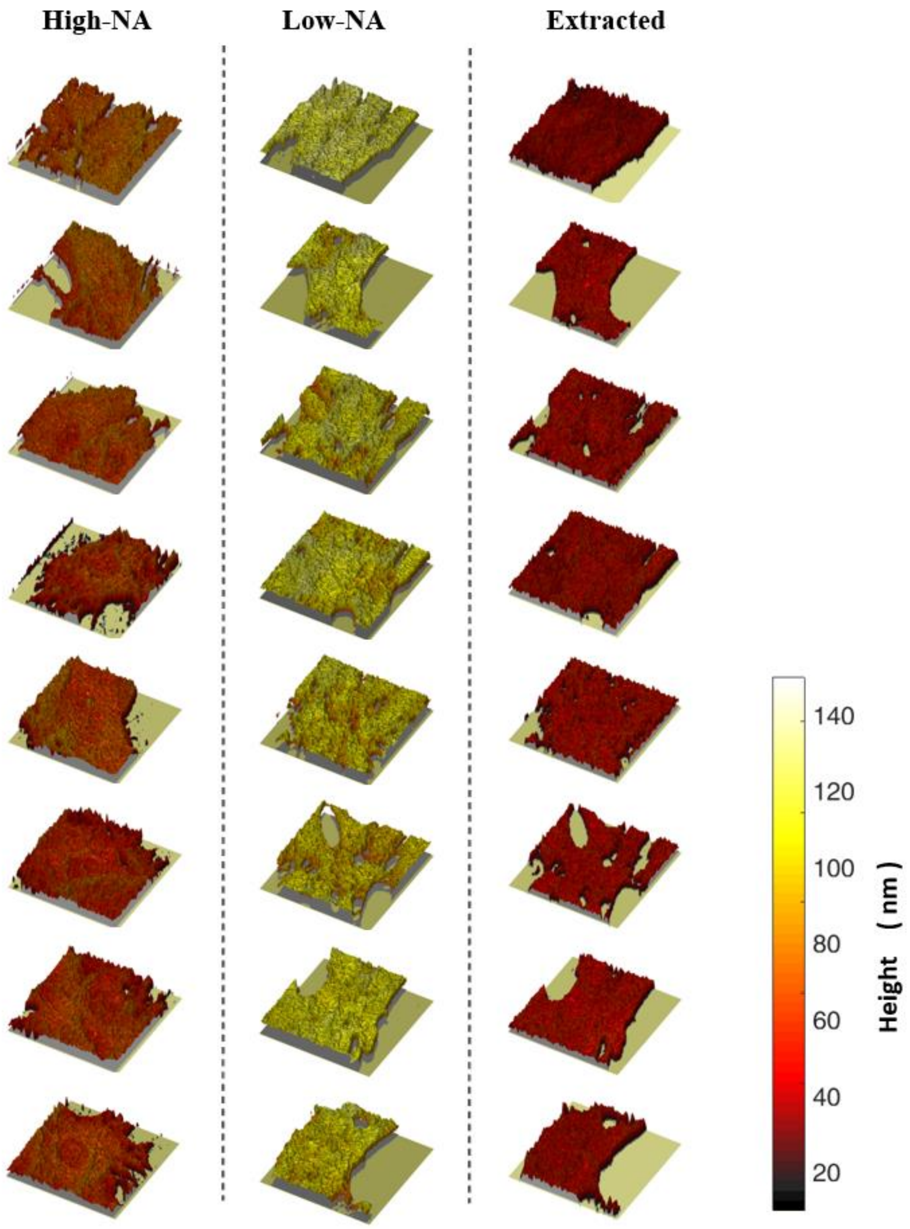

Supplementary Figure 10

Side-by-side comparison of topographic maps of the high-NA, low-NA, and extracted groups

Topographic images of cell-substrate distance calculated from the three different groups of FLIM images shown in Supplementary Fig. 8: high-NA, low-NA, and extracted-image groups. We used the lifetime-to-distance (LTD) curve shown in Fig. 2g, which is obtained by the CPS model (Methods). The color bar on the right side represents heights ranging from 10 nm to 150 nm.

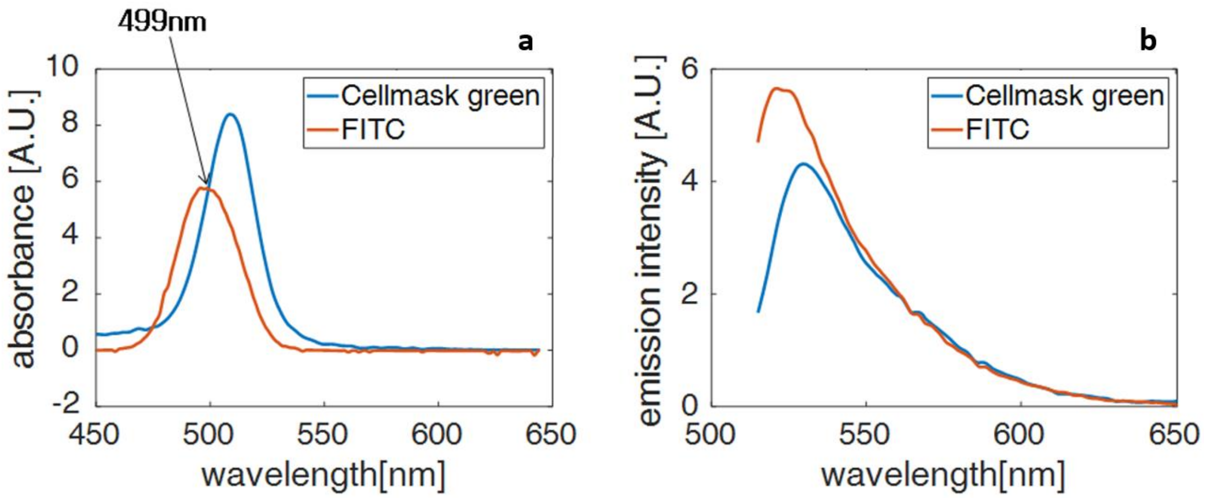

Supplementary Figure 11

Absorption and emission spectra of CellMask™ Green (CMG) and fluorescein isothiocyanate (FITC) in DPBS

(a) Measured absorption spectra of FITC and CMG. **(b)** Measured emission spectra of FITC and CMG when they are excited at 499 nm wavelength. We used a fluorescence spectrometer (LS-55, PerkinElmer Inc.) to obtain these data. The quantum yield (QY) of CMG is required to obtain the lifetime-to-distance (LTD) conversion curve shown in Fig.2g. Since we could not find the QY of the CMG in the literature, we have calculated it by using the known QY (0.93) of FITC[3] (Supplementary Note 1). Fluorescence emission spectra of FITC and CMG in (b) are used in this calculation.

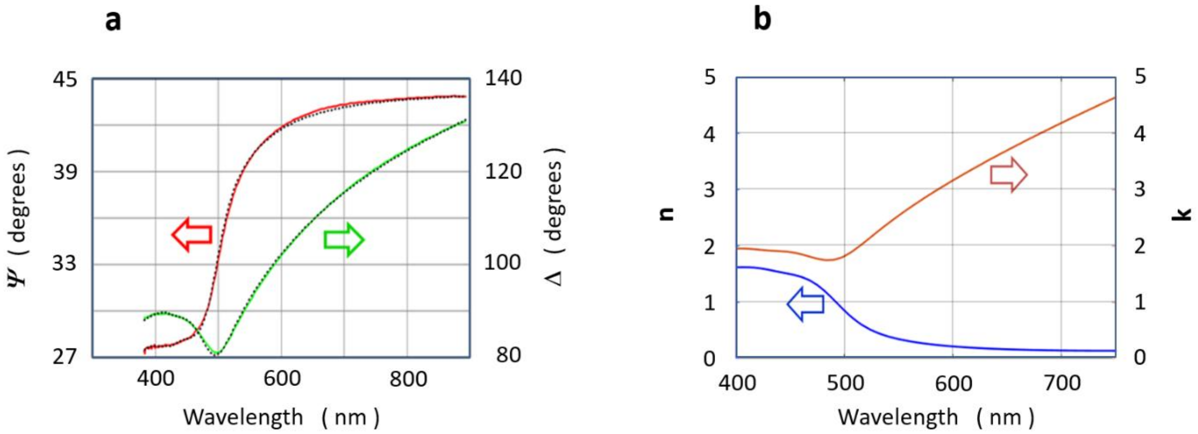

Supplementary Figure 12

Thickness and refractive index measurements for an Au film with an ellipsometer

The thickness and the optical constants of the Au film used in MIET imaging are needed to obtain the lifetime-to-distance (LTD) conversion curve. A spectroscopic ellipsometer (alpha-SE, J.A. Woollam Co.) is used to measure the optical constants and the thickness of Au film we used for MIET imaging. **(a)** Raw spectroscopic ellipsometry data of a thin Au film on a coverslip made of BK7 glass: $\frac{r_p}{r_s} = tan\Psi \cdot e^{i\Delta}$. $tan\Psi$ is the amplitude ratio of Fresnel's reflection coefficients for P and S polarized lights, and Δ is the phase difference between them. $\Psi(\lambda)$ and $\Delta(\lambda)$ are measured in a spectral range from λ = 380 nm to λ = 900 nm using three incident angles of 65°, 70°, and 75°. **(b)** Calculated dispersion curves for the complex index of refraction $n_m(\lambda) = n(\lambda) + ik(\lambda)$. The thickness and the complex refractive index of a film can be calculated by fitting the data shown in (a) with the Fresnel's equations for multiple reflected lights by a thin film on a substrate[4]. We obtained $d = 51.6\ nm$ for the thickness of the Au film, and $n_m = 0.23 + 2.22i$ for the complex index of refraction of Au film at $\lambda = 530\ nm$, the peak emission wavelength of CellMask™ Green.

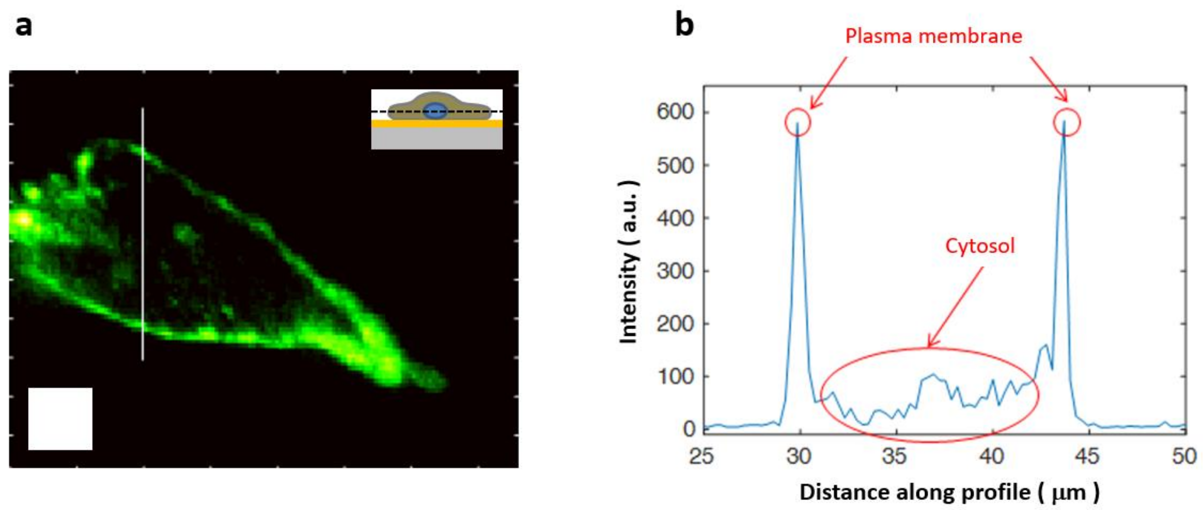

Supplementary Figure 13

Verification of cellular internalization of CellMask™ Green with confocal microscopy

When CellMask™ Green (CMG) was used to visualize plasma membranes, a small amount of CMG may be internalized. If there exist internalized CMG within 125 nm distance from the metal layer in our proposed method, it can be an error source. However, any internalized CMG within 0.62 nm (the axial resolution of the high-NA objective) from a metal film can generate errors in conventional MIET with a high-NA objective. We have imaged internalized CMG with our confocal microscopy system. We took 20 axial sectioning images while shifting a sample stage vertically along the z-axis with a step size of 500 nm by using a motorized stage. **(a)** An axial sectioning confocal fluorescence image of a HAEC stained with CMG. It is measured with an objective of NA = 1.49 and a confocal pinhole of 1 Airy unit, which produces the axial sectioning resolution to be 0.62 μm. To visualize the cell's cytosol, we set the sectioning height of the image to 1 μm above the gold film, which is in the middle of the apical and the basal membranes of the cell (subset at the upper right corner). The bright boundary of a cell in the image is where the axial sectioning plane of the confocal microscopy hits the cell's plasma membrane. Area within the cell boundary is the cell's cytosol, and non-zero fluorescence intensities in this area are due to the internalized CMG. A small white box at the lower-left corner is a square of 5 μm side-length. **(b)** Line scan intensity profile along the white line in (a). Two peaks near 30 μm and 44 μm along the horizontal axis are from the cell's plasma membrane. It shows that the fluorescence intensities from the cytosol are about 10 % of the peak intensity from the membrane. As the FWHM of axial PSF for the high-NA objective is 0.62 μm, while the sectioning height of the image from the gold film is only 1 μm, it is difficult to quantify the average amount of internalized fluorophore from this sectioning fluorescence intensity. Non-zero intensities between these two peaks are evidence of internalized fluorophores in the cytosol of the cell.

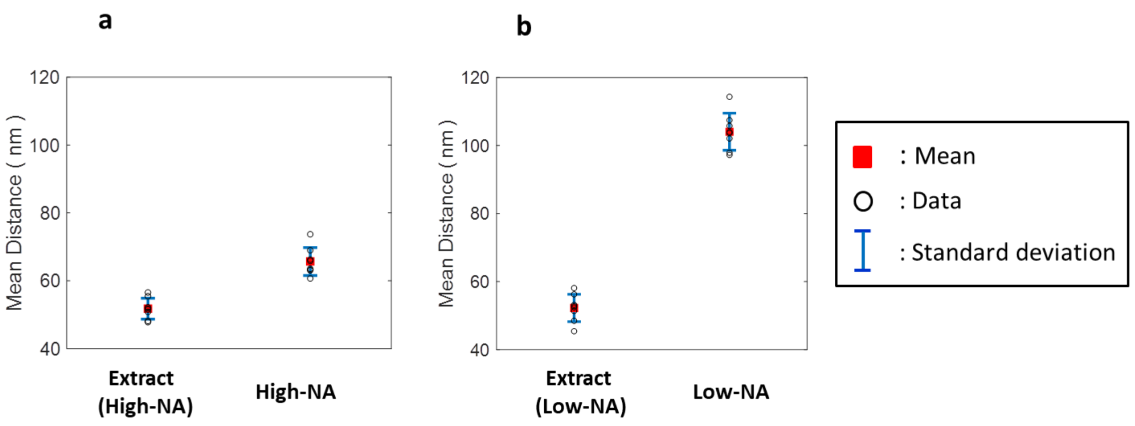

Supplementary Figure 14

Comparison of cell-substrate distances before and after biexponential signal analysis

(a) Mean cell-substrate distances before and after the biexponential signal analysis for the high-NA image groups shown in Supplementary Fig. 10. Extracted distances were calculated by our proposed biexponential signal analysis. The mean cell-substrate distance is reduced from 63 nm to 50 nm when the lower value of extracted lifetime components is used for each pixel in distance measurement. **(b)** Mean cell-substrate distances before and after the biexponential signal analysis for the low-NA image groups shown in Supplementary Fig. 10. The mean cell-substrate distance is reduced from 104 nm to 52 nm when the lower value of extracted lifetime components is used for each pixel.

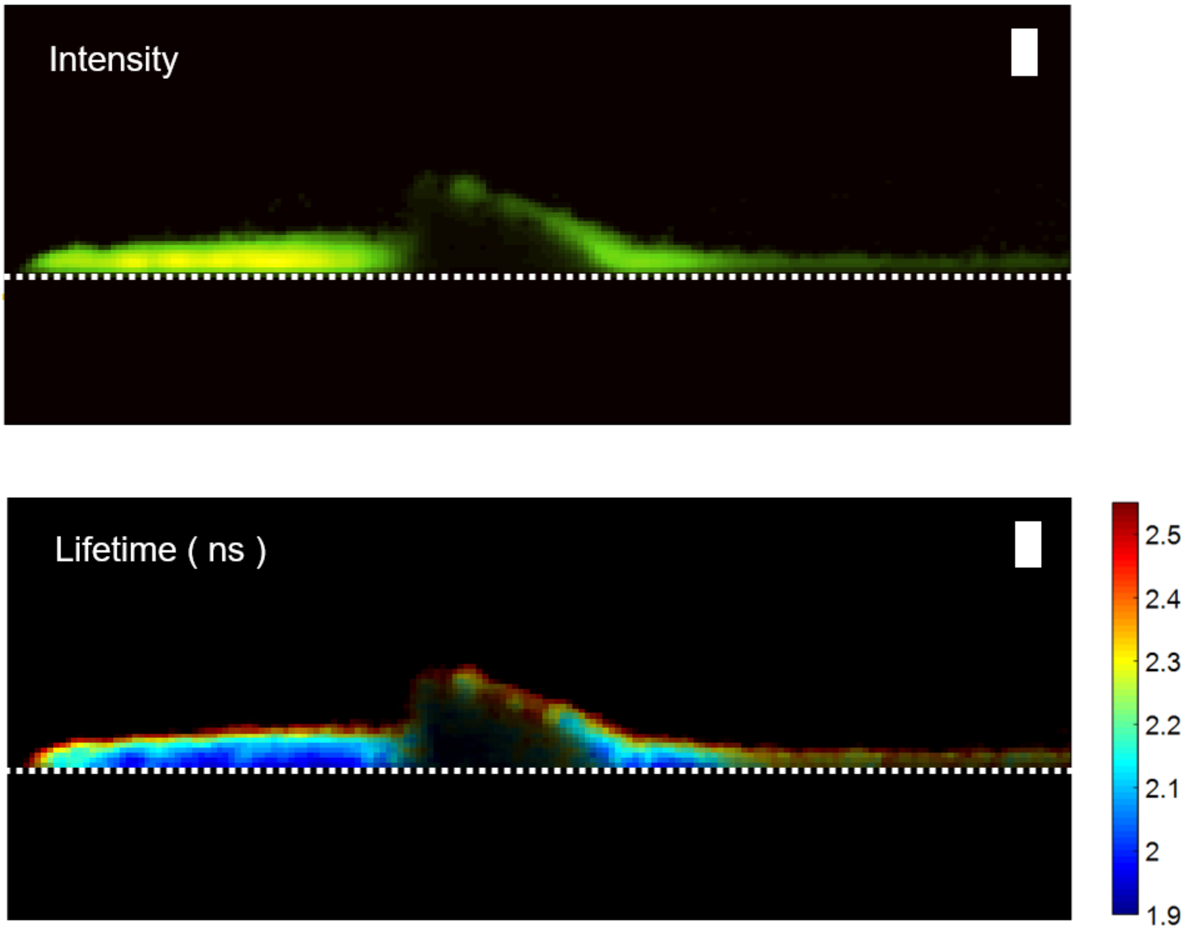

Supplementary Figure 15

Confocal fluorescence intensity and lifetime images of a HAEC on gold film (50nm) on the xz-plane

The HAEC is labeled with CMG and measured with the high-NA objective (NA = 1.49). The PSF of our confocal microscope with the high-NA objective is shown in Supplementary Figure 2. The upper figure is an intensity image, and the lower one is the corresponding lifetime image. Two white rectangular boxes in the upper right corners show 1 μm x 1 μm scale. The image size is 40 μm x 9 μm. The lifetimes of apical membrane range 2.3 ~ 2.5 ns, whereas those of basal membrane are 1.9 ~ 2.0 ns.

Supplementary Note 1

Calculation of quantum yield (QY) for CellMask™ Green (CMG)

The quantum yield of an unknown fluorophore can be obtained from a known fluorophore based on the following simple equation[5].

$$\frac{QY}{QY_r} = \frac{I}{I_r}\frac{\left(1-10^{A_r}\right)}{\left(1-10^{A}\right)}\frac{n^2}{n_r^2} \qquad (1)$$

$\boldsymbol{QY}$ and $\boldsymbol{QY_r}$ are quantum yields of an unknown and a known reference fluorophore, $\boldsymbol{I}$ and $\boldsymbol{I_r}$ are integrated fluorescence emission spectra, $\boldsymbol{A}$ and $\boldsymbol{A_r}$are absorbances, $\boldsymbol{n}$ and $\boldsymbol{n_r}$ are refractive indices of them at a given excitation wavelength. The reference fluorophore is FITC, and the unknown fluorophore is CMG in this case. Since both fluorophores were in DPBS whose refractive index is identical to that of NaOH used in QY measurement of FITC[3], we have $\boldsymbol{n} = \boldsymbol{n_r}$. In data shown in Supplementary Fig. 11(a), the absorbance of FITC and CMG are identical at 499 nm wavelength. This can simplify eq.(1) with $\left(\mathbf{1}-\mathbf{10}^{A_r}\right) = \left(\mathbf{1}-\mathbf{10}^{A}\right)$. Supplementary Fig. 11(b) shows the measured emission spectra of FITC and CMG when both fluorophores are excited at 499 nm wavelength. We have obtained $\boldsymbol{I}/\boldsymbol{I_r} = \mathbf{0.9}$ by integrating the emission spectra of FITC and CMG shown in Supplementary Fig. 11(b). From eq. (1) we obtained that the QY of CMG is 0.84 in DPBS, while CMG's fluorescence lifetime was measured to be 2.74 ns in DPBS. Since the fluorescence lifetime of CMG is reduced to 2.43 ns when embedded within the membrane of a HAEC (Supplementary Fig. 4), we deduce that the QY of CMG is reduced to 0.74 when it is embedded within the membrane.

Supplementary References

1. Wilson, T. Resolution and optical sectioning in the confocal microscope. *Journal of microscopy* **244**, 113-121 (2011).
2. Won, Y.J., Han, W.-T. & Kim, D.Y. Precision and accuracy of the analog mean-delay method for high-speed fluorescence lifetime measurement. *JOSA A* **28**, 2026-2032 (2011).
3. Weber, G. & Teale, F. Determination of the absolute quantum yield of fluorescent solutions. *Transactions of the Faraday Society* **53**, 646-655 (1957).
4. Tompkins, H.G. & Hilfiker, J. Spectroscopic Ellipsometry. *Practical Application to Thin Film Characterization* (2016).
5. Berndt, M., Lorenz, M., Enderlein, J.r. & Diez, S. Axial nanometer distances measured by fluorescence lifetime imaging microscopy. *Nano letters* **10**, 1497-1500 (2010).
