# Supplementary material for: Large field-of-view nanometer-sectioning microscopy by using metal-induced energy transfer and biexponential lifetime analysis
Source: Commun Biol. 2021 Jan 19;4:91. doi: 10.1038/s42003-020-01628-3 (PMC7815909; doi:10.1038/s42003-020-01628-3)
Supplement: Supplementary file 2 — Description of Additional Supplementary Files [file 42003_2020_1628_MOESM2_ESM.pdf]

Description of Additional Supplementary Files

File Name: Supplementary Data 1
Description: The mean values of the measured cell-substrate distances from high-NA, low-NA, and extracted-image groups. These data are presented in Fig.2.i.
